# COGNITIVE DECLINE AMONG OLDER ADULTS FOLLOWING TRAUMATIC BRAIN INJURY

**DOI:** 10.1093/geroni/igae098.4300

**Published:** 2024-12-31

**Authors:** Jennifer Albrecht

**Affiliations:** University of Maryland School of Medicine, Baltimore, Maryland, United States

## Abstract

Traumatic brain injury (TBI) is a risk factor for Alzheimer’s disease and related dementias. However, evidence for this association among older adults is less robust, and further studies are needed to better understand cognitive recovery following TBI in this population. Retro-TBI is an ongoing prospective cohort study of adults aged 65+ diagnosed with TBI at a Level 1 trauma center. In the current study, we assessed four cognitive domains (memory, language, attention, and visuospatial/constructional) at 2 weeks, 3 and 6 months post-TBI using the Repeatable Battery for the Assessment of Neuropsychological Status (RBANS). We calculated the slope of the RBANS total age-adjusted scaled scores for each participant with ≥2 assessments (n=42) over days since TBI. Participants with a negative slope were considered to have cognitive decline. We compared participant characteristics between those with and without cognitive decline using Student’s t-test or Fisher’s exact test. Average age was 73.9 (standard deviation 7.8) years and 47.6% were female. Findings indicated that 31.0% (13/42) of participants had cognitive decline and were more likely to report a history of depression (61.5% vs. 13.8%, p=.003) and live alone (76.9% vs 34.5%, p=.02) than those without cognitive decline. The cognitive decline group was also more likely to experience decline in visuospatial/constructional (61.5% vs. 24.1%, p=.04) and immediate memory skills (61.5% vs. 27.6%, p=.047) compared to those without cognitive decline. Preliminary findings from this ongoing study suggest that one-third of older adults experience cognitive decline over 6 months post-TBI, with immediate memory and visuospatial skills particularly impacted.

